# Effect of Bone Regeneration with Mineralized Plasmatic Matrix for Implant Placement in Aesthetic Zone

**DOI:** 10.1155/2017/2639564

**Published:** 2017-03-28

**Authors:** Khadija Amine, Amina Gharibi, Azeddine Hsaine, Jamila Kissa

**Affiliations:** ^1^Periodontics Department, Casablanca Dental School, Casablanca, Morocco; ^2^Prosthodontics, Casablanca Dental School, Casablanca, Morocco

## Abstract

Bone volume is one of the key factors to be considered when evaluating implant placement. When the bone volume is insufficient, implant placement could be conditioned by the necessity of preforming bone grafting procedures to compensate bone loss. Various grafting procedures can be used with different bone substitute. Mineralized Plasmatic Matrix (MPM) is one of these grafting materials, used to maintain or regenerate the socket's volume. In MPM, the autologous blood products highly concentrated in platelets and fibrin in a liquid state are combined with a bone substitute. The fibrin can become bound to bone particles. The filling material is easy to shape and a PRF-type membrane is also generated. In the present case we report the application of MPM in two sites presenting bone crest defects when placing implant in those areas.

## 1. Background 

Soft and hard tissue defects create an anatomically less favourable context for ideal implant placement. Alveolar bone defects occur due to periodontitis, trauma, tumors, or resorption following tooth extraction [1].

To achieve optimum treatment outcome with dental implants, sufficient bone should be available to support and stabilize them [2, 3].

Reconstruction of the alveolar bone through a variety of regenerative surgical procedures had become predictable [1, 4]. Autogenous, allogenic, and tissue engineered [5, 6] bone grafts are successfully used. The success rates in autogenous bone graft are from 73.8% to 100% and 95.3–100%, in allogenic bone grafts [1].

It may be necessary prior to implant placement or simultaneously at the time of implant surgery to provide a restoration with a good long-term prognosis [7].

Grafting materials and absorbable membranes [8] were also proposed for postextraction alveolar ridge preservation. The introduction of protein therapy in regenerative procedures could overcome the use of barrier membranes in certain cases making grafting procedures easier.

The Mineralized Plasmatic Matrix (MPM) is an autologous blood product highly concentrated in platelets and fibrin in a liquid state combined with a bone substitute. The fibrin can become bound to bone particles.

The filling material is easy to shape and a PRF-type membrane is also generated [9].

The aim of this case report is to demonstrate that a one-step surgical procedure using a MPM graft around implant is suitable and successful in areas that have a narrow ridge.

## 2. Case Presentation 

**Figure 1:**
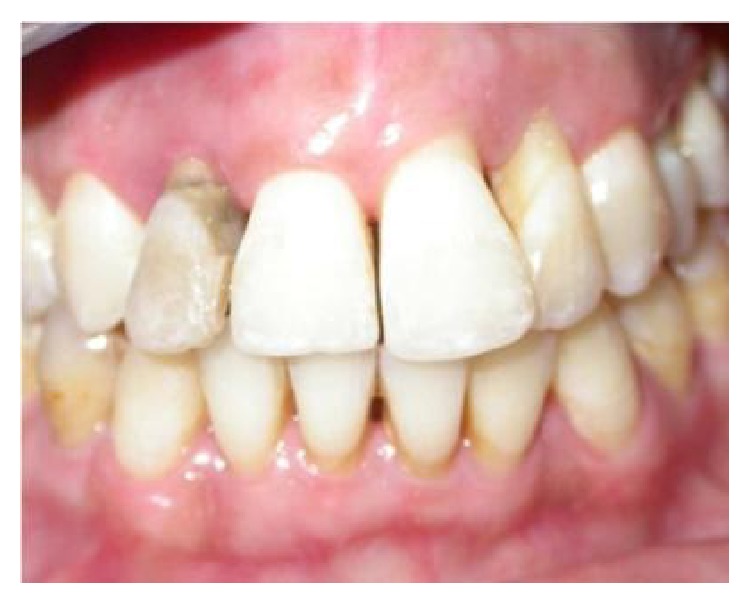
Anterior clinical view showing the extrusion of the teeth 12, 22. Aggressive periodontitis.

**Figure 2:**
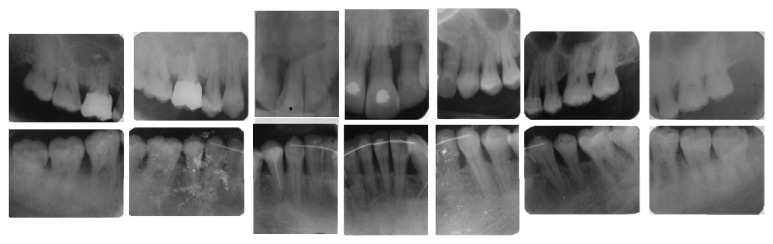
Full mouth radiographs demonstrating generalized severe horizontal and angular bone loss.

A 34-year-old female in good general health, nonsmoker, with a history of aggressive periodontitis was referred for evaluation and treatment (Figure 1). The initial periodontal therapy was done. The maxillary incisors had severe bone loss with hopeless prognosis (Figure 2). Hence, we decided to extract the four incisors.

## 3. Case Management 

Periodontal therapy including scaling and root planning was performed two months prior to the surgery.

For financial reasons, side by side surgery was decided.

### 3.1. Stage 1: Site of 12

First the extraction of the 12 was done.

**Figure 3:**
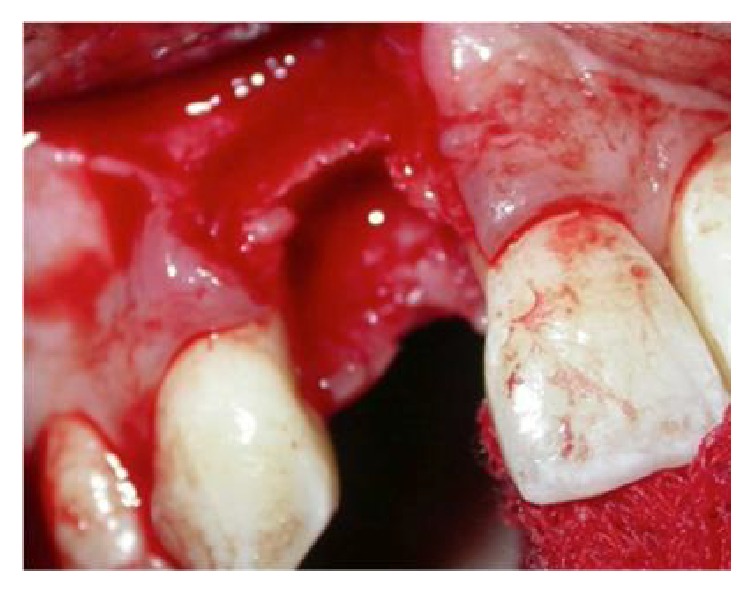
Vertical and horizontal bone defect after extraction of 12.

**Figure 4:**
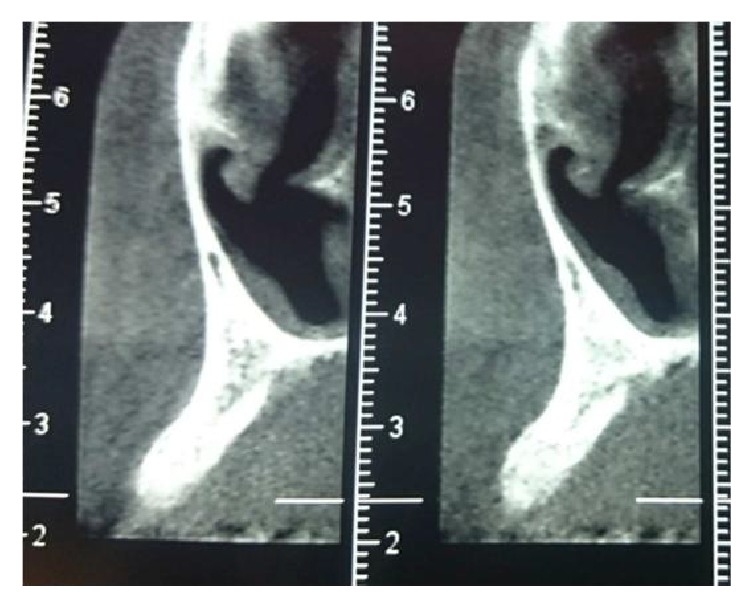
Insufficient bone.

After 4 months of healing, horizontal and vertical postextraction defect was clinically noticed (Figure 3) and the cone beam computed tomography revealed that the bone dimension was not enough for an implant placement (Figure 4).

A protein technique using MPM (Mineralized Plasmatic Matrix) was performed for horizontal augmentation and an implant was placed.

4 tubes of 9 mL of patient's blood were taken, to prepare the MPM [10]. The venous blood was placed into the centrifugation machine to separate the red blood cells from the platelets for 8 min at 2700 RPM. The result obtained after the centrifugation was two layers: a yellow plasma liquid on the top of the tube separated from the red blood cells in its bottom.

**Figure 5:**
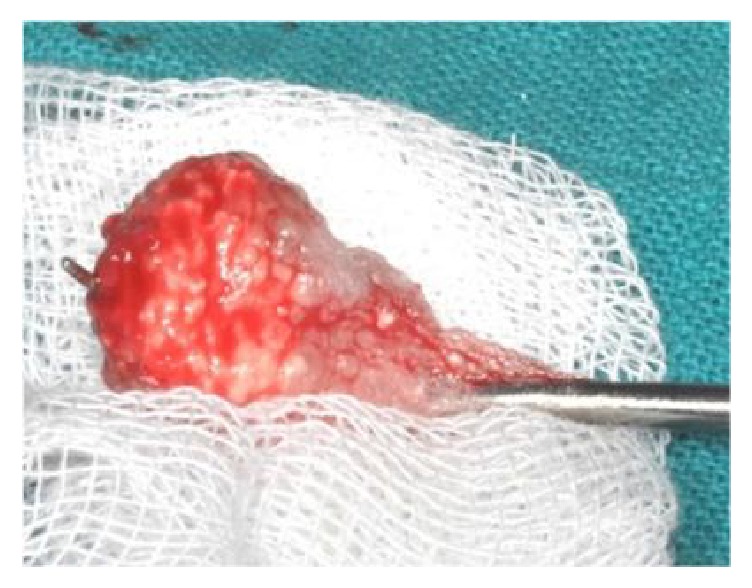
The MPM is deposited in a sterile gauze.

**Figure 6:**
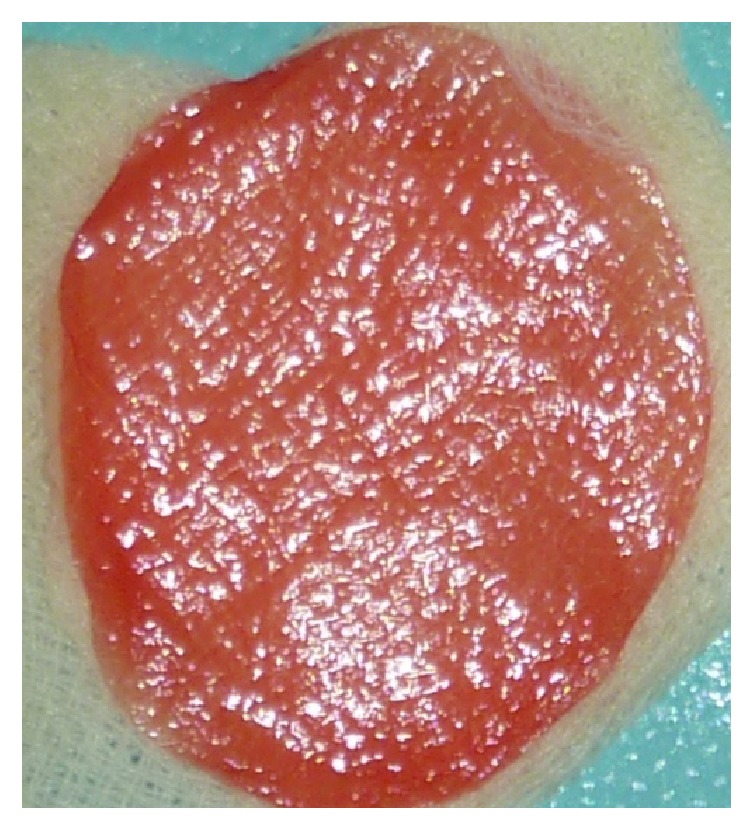
The MPM is compressed to obtain a PRF like membrane.

The yellow part was collected using a syringe and added to a cup that contains the bone grafting material (HA 30% + *β*-TCP 70%) and autologous bone. The whole preparation was mixed for few seconds and the MPM was obtained (Figures 5 and 6).

**Figure 7:**
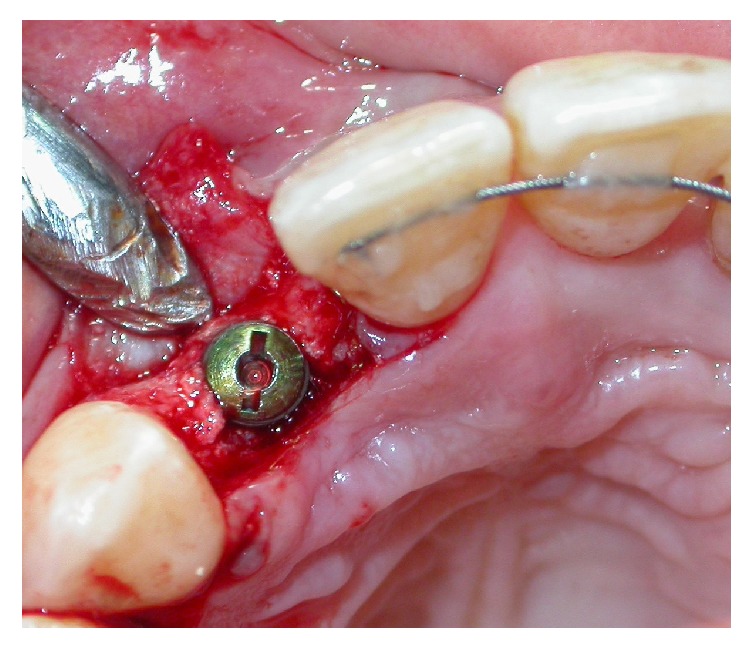
Implant placement in a narrow bone crest.

**Figure 8:**
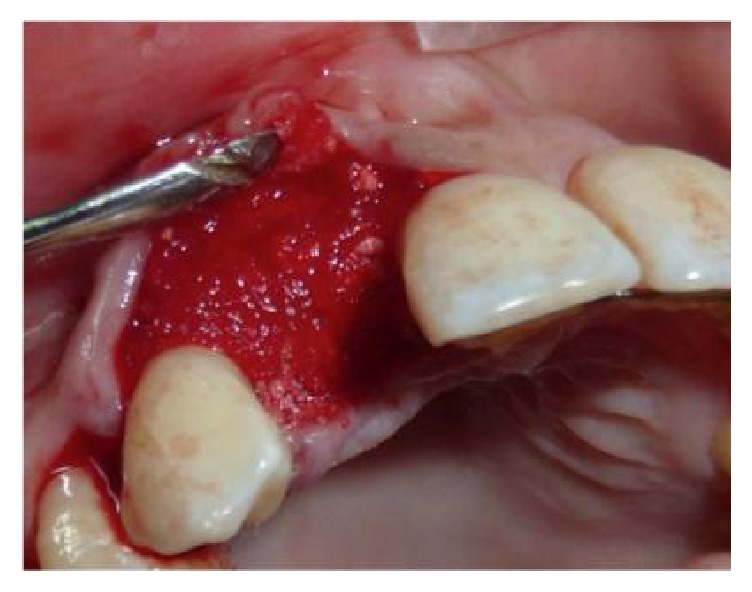
The MPM is placed around the implant.

**Figure 9:**
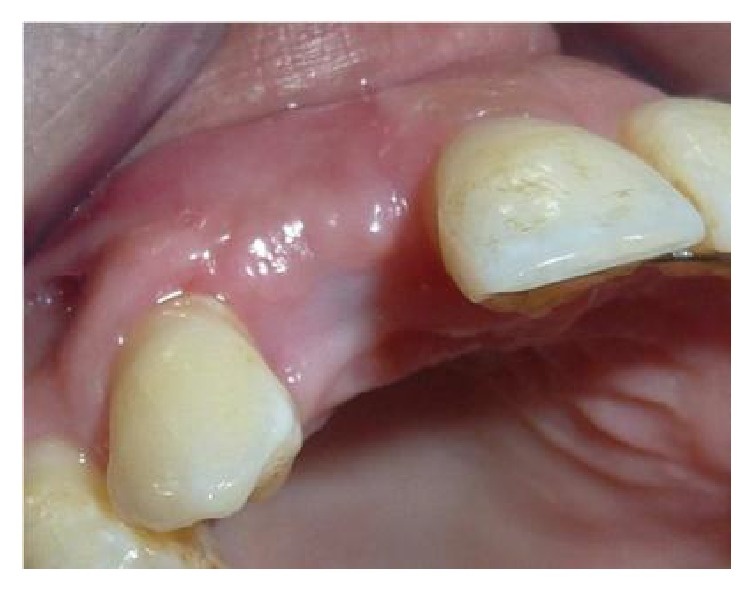
Occlusal view after 4 months' healing showing a good vestibular volume.

**Figure 10:**
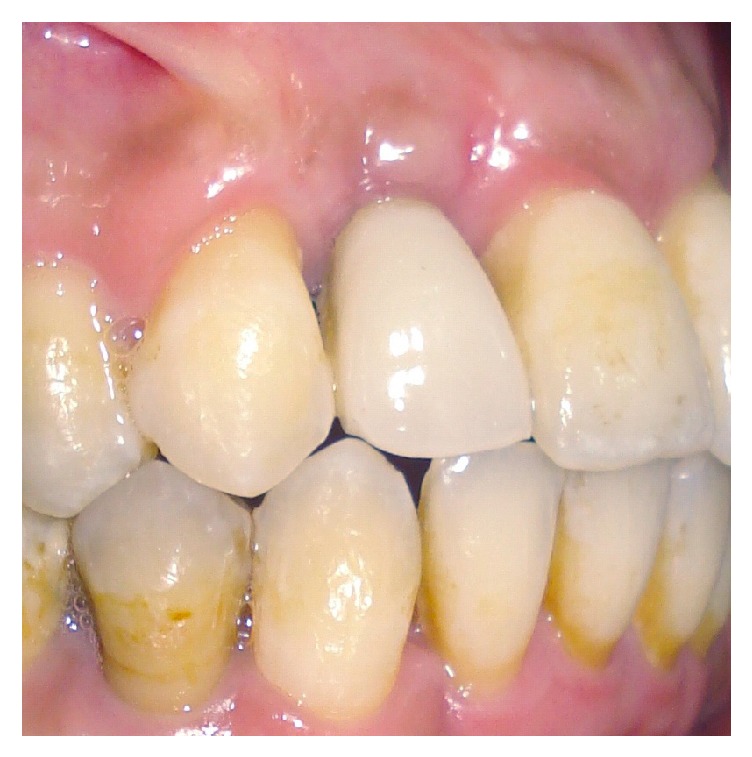
The temporary crown on the 12.

**Figure 11:**
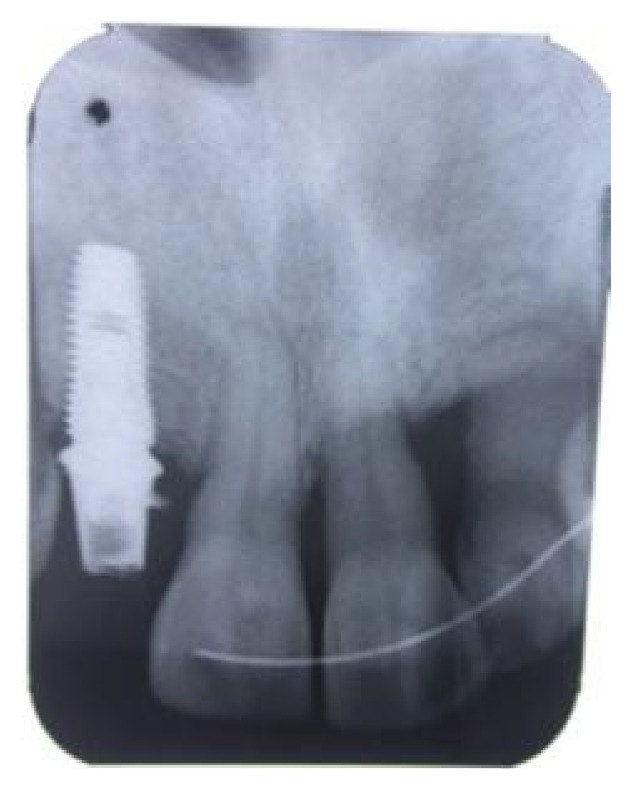
A radiograph showing the implant placement.

After placing the implant (Figure 7), the MPM and its membrane were placed to correct the bone defect. The implant was completely covered by the modified MPM and sutures were performed (Figure 8). After 4 months of healing a good vestibular volume was acquired (Figure 9). Then the second-stage surgery was done and the temporary crown was placed on the 12 (Figures 10 and 11).

### 3.2. Stage 2: Site of 22

**Figure 12:**
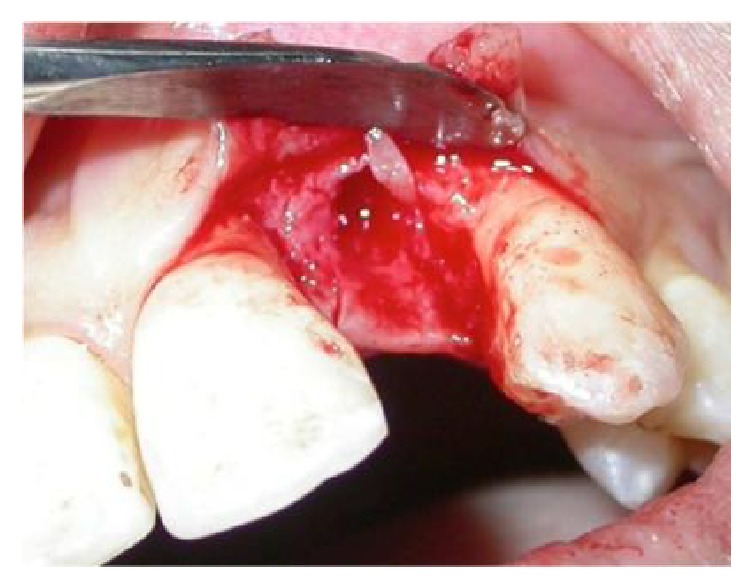
Osseous bone defect after extraction of the 22.

**Figure 13:**
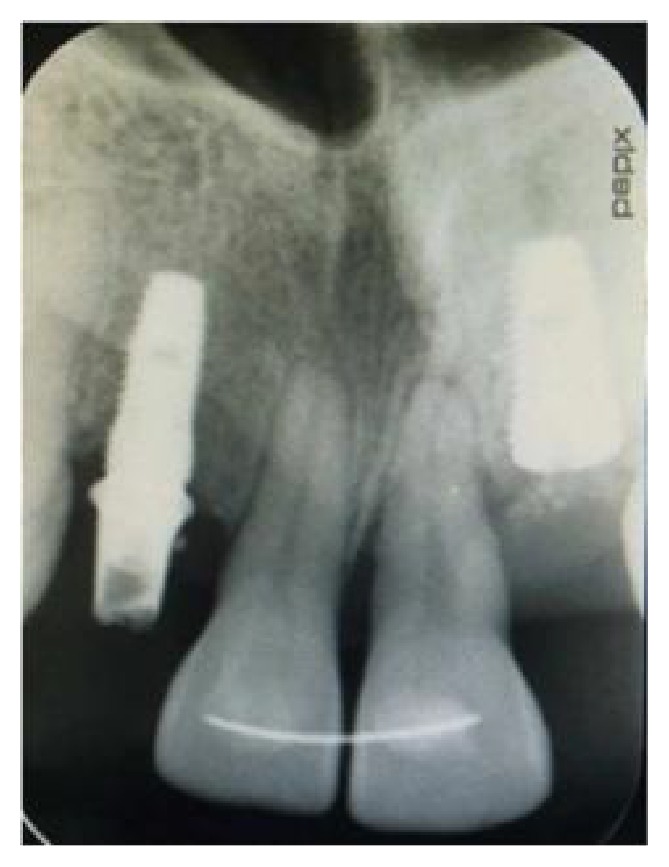
A radiograph showing the 2 implants replacing the teeth 12, 22.

After the extraction of the 22 (Figure 12), immediate implant placement was performed, and MPM was placed around the site. A control radiograph showing the position of the implants was taken (Figure 13).

### 3.3. Stage 3: Final Bridge

The extraction of 11 and 21 was performed after four months and a bridge was done (Figure 15).

## 4. Case Outcome 

**Figure 14:**
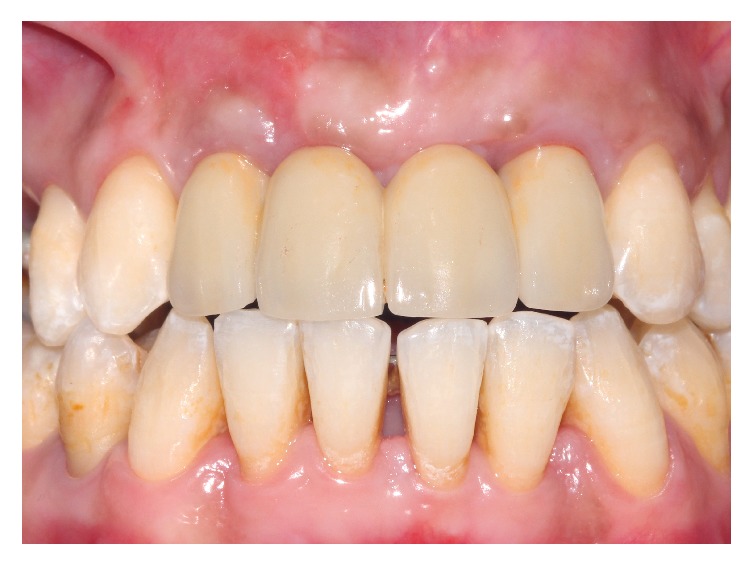
The final prosthetic rehabilitation.

**Figure 15:**
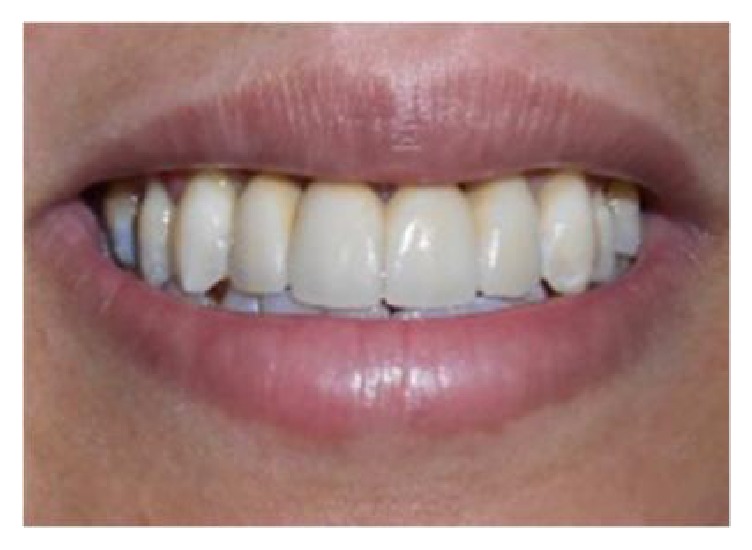
Final esthetic and functional rehabilitation.

This procedure allowed better correction of the horizontal vestibular defect and an aesthetic soft tissue improvement (Figures 14 and 15).

## 5. Discussion 

To achieve an optimal esthetic outcome, implants must be placed in an optimal position and inclination (de Lange 1995; Phillips and Kois 1998). When there are bone resorptions due to periodontitis, osseous regeneration might be necessary.

The success of the reconstructive procedures is influenced by the span of the edentulous ridge [11] and the amount of attachment on the neighbouring teeth [12]. Although autologous onlay bone graft techniques have been considered as gold standard, for horizontal augmentation [13], donor sites morbidity [14] associated with block grafts have turned attention to the use of other bone graft materials.

The use of MPM in periodontology and implant therapy seems to have a great impact in the outcome of the grafting surgery. It allows the conduction and homogenization of the grafting materials [15].

The MPM is a natural evolution of the platelet rich plasma [10]. PRP is an autologous modification of fibrin glue and is used to deliver the growth factors in high concentration to the bone site. PDGF and TGF-*β* are the wound healing substances that have shown to play an important role in the healing of bone. One of the highest concentrations of PDGF and TGF-*β* in the body is found within the blood platelets [16].

Marx et al. [17] claimed that platelet concentration can increase from an average of 232.000 to 786.000 per microliter of blood. The authors used combination of PDGF gel with bone grafts in the reconstructive osseous surgeries and observed significant bone regeneration and increased bone density and maturation rates in 40 osseous defects [17]. Furthermore, the interesting part in the modified MPM is the mineral fraction, which is either autologous bone or any other bone graft or bone substitute.

During manipulation, the retention in the fibrous mesh of the bone fragments or the grafting material conserves its cohesion and avoids its departure away from the recipient bed [10].

Therefore, the use of MPM in periodontology and implant therapy has a great impact in the outcome of the grafting surgery because it enhances transport of the material by securing its implementation [18].

In this case, this new procedure seems to provide a more predictable rehabilitation of the hard and soft tissues.

However, more well designed and properly controlled comparative studies are needed to provide solid evidence of MPM capacity to improve wound healing, bone augmentation procedures, and soft tissue reconstruction.
